# Regulation of the DNA Damage Response to DSBs by Post-Translational Modifications

**DOI:** 10.2174/138920210791110979

**Published:** 2010-05

**Authors:** C. Oberle, C. Blattner

**Affiliations:** Karlsruher Institute of Technology, Institute of Toxicology and Genetics, Karlsruhe PO-Box 3640, 76021 Karlsruhe, Germany

**Keywords:** DNA double strand breaks, homologous recombination, non-homologous endjoining, cell cycle arrest, post-translational modifications.

## Abstract

Damage to the genetic material can affect cellular function in many ways. Therefore, maintenance of the genetic integrity is of primary importance for all cells. Upon DNA damage, cells respond immediately with proliferation arrest and repair of the lesion or apoptosis. All these consequences require recognition of the lesion and transduction of the information to effector systems. The accomplishment of DNA repair, but also of cell cycle arrest and apoptosis furthermore requires protein-protein interactions and the formation of larger protein complexes. More recent research shows that the formation of many of these aggregates depends on post-translational modifications. In this article, we have summarized the different cellular events in response to a DNA double strand break, the most severe lesion of the DNA.

## INTRODUCTION

The integrity of cellular DNA is threatened every day by exogenous factors like UV-light, ionizing radiation or environmental and therapeutic chemicals, as well as by endogenous conditions such as DNA replication, meiotic recombination or programmed rearrangements [reviewed in: 1-4]. These DNA lesions can result in somatic mutations, cell death, genomic instability and carcinogenesis. Accordingly, maintenance of genomic integrity represents one of the most important challenges for cellular organisms. To ensure functionality despite recurring insults, cells have developed several strategies to handle DNA lesions which are in principle prohibition of further cell divisions, repair of the lesions and elimination of cells with damaged DNA.

The repair of DNA lesions is a complex process. Moreover, diverse damages of the DNA that result from exposure to various adverse agents are fixed by different repair programs. Single strand breaks and damaged nucleotides that can result from oxidation, alkylation, hydrolysis, deamination or exposure to ionizing rays are replaced through base excision repair (BER), a mechanism that uses the sister strand as a template to direct *de novo* synthesis of the damaged strand around the lesion [reviewed in: 5]. Photoproducts and bulky DNA adducts that result e.g. from covalent binding of chemicals to DNA bases or exposure to UV-light are fixed by the nucleotide excision repair (NER) pathway [reviewed in: 6]. The mismatch repair (MMR) pathway, on the other hand, eliminates mispaired nucleotides that can arise during DNA replication and recombination [reviewed in: 7]. Ionizing radiation and replication in the presence of DNA lesions lead to the generation of DNA double strand breaks (DSBs), which are mainly fixed by two different repair mechanisms: homologous recombination (HR) and non-homologous endjoining (NHEJ).

While relatively rare, DSBs are the most severe DNA lesion since repair is difficult und the original state is seldom fully reconstituted. Moreover, we still understand only little about the response to DSBs, although a large number of the proteins that are involved have been identified. However, it is still ill-defined how these proteins are recruited to the site of the lesion and how the different factors interact and communicate with each other to repair the damage and to guard the genome. In this review we will summarize the cellular response to DSBs. Hereby, we will particularly focus on signal transduction processes that occur during DNA damage recognition and repair.

Post-translational modifications of existing molecules play an important role in the cellular response to DSBs since they provide a means to alter the activity of a given protein without the necessity of *de novo* protein synthesis. Thus, the properties of a protein can be changed rapidly and without the need to transcribe damaged DNA which could be grossly hindered by the lesion or may result in non-functional protein products. While already addition or removal of small chemical groups modifies the characteristics of a target protein dramatically, small proteins of the family of ubiquitin-like proteins are also frequently attached to molecules involved in different cellular processes, to alter their activities or to extend their repertoire of protein-protein interactions. The most common post-translational modifications, also during the response to DSBs, are phosphorylation, acetylation, methylation, ubiquitination, SUMOylation and NED-Dylation. All these modifications are reversible and, accordingly, regulated by two types of enzymes. Phosphorylation is, for example, performed by kinases that covalently link a phosphate group onto a serine, threonine or tyrosine of the target protein [reviewed in: 8]. Phosphatases reverse this alteration by removing the phosphate group [reviewed in: 9]. Phosphorylation frequently results in a conformational change of the modified protein due to the introduction of a negative charge and may result in activation or inactivation of an enzyme. Alternatively, it can alter the capacity of a target protein to associate with other cellular factors [reviewed in: 8]. The property of a protein can also be changed by reversible addition of acetyl- or methyl-groups, a process that is similar to the attachment of phosphate groups [reviewed in: 10, 11]. Acetyl-groups are attached to lysine residues in the target protein by acetyltransferases and removed by deacetylases [reviewed in: 10]. Lysines as well as arginines can further be modified by addition of a methyl-group, which is catalyzed by methyltransferases [reviewed in: 11]. The functional outcome of acetylation and methylation depends on the target protein and the position of the acetylated/methylated lysine in the molecule. This type of modification affects frequently protein-protein interactions, protein-DNA interactions or protein degradation (due to competition with the ubiquitination machinery for the same lysine) [12, 13].

The function of certain proteins can further be influenced by covalent addition of members of the family of small ubiquitin-like proteins. The first protein of this group that was identified was ubiquitin itself, a protein of 76-amino acids that is ubiquitously expressed from yeast to man [reviewed in: 14]. Ubiquitin is implicated in a multitude of cellular processes like regulation of the cell cycle, DNA replication, DNA repair, stress response, apoptosis, signal transduction or in the biogenesis of ribosomes, nucleosomes and peroxisomes [reviewed in: 15-17]. The covalent ligation of ubiquitin is an extremely conserved process and involves several enzymes and accessory proteins that are generally highly conserved across different species [reviewed in: 15]. Moreover, the attachment of ubiquitin onto a target protein ranges from the attachment of only one ubiquitin molecule to one lysine (monoubiquitination) over the addition of one ubiquitin to several lysine residues within the same protein (multiubiquitination) to the formation of chains of ubiquitin molecules on one individual lysine (polyubiquitination) [reviewed in: 18]. Since the ubiquitin protein itself possesses 7 lysine residues (lysine 6, lysine 11, lysine 29, lysine 31, lysine 33, lysine 48, lysine 63), which all can serve as an anchor for ubiquitination, a variety of chains can be formed that control different properties [reviewed in: 19, 20]. Polyubiquitination of lysine 48 is the prototype for chains that lead to proteosomal degradation, although more recent reports showed that polyubiquitination of lysine 11 and eventually also of lysine 63 can target substrate proteins for destruction [21, reviewed in: 19, 20]. Proteins that are polyubiquitinated at lysine 63 are frequently implicated in DNA repair, while chain formation at lysine 29 seems to label their targets for lysosomal degradation [22, 23]. In addition, polyubiquitin chains are not necessarily homotypic. Chains, containing two types of linkages (lysine 6/11, lysine 27/29, lysine 29/48; lysine 29/33) have also been observed [24]. Like phosphorylation or acetylation, the modification of proteins with ubiquitin is reversible. Specific deubiquitinating enzymes (DUBs) hydrolyze the isopeptide bond between ubiquitin and its substrate and thus remove the ubiquitin molecule or chain from its target [reviewed in: 25].

Another member of the ubiquitin family is SUMO (small ubiquitin-like modifier), a small polypeptide of 10-11 kDa [reviewed in: 26, 27]. SUMOylation is frequently involved in the control of recombination, DNA repair, nucleocytoplasmatic transport, chromosomal dynamics, meiosis and mitosis and the maintenance of genomic stability [reviewed in: 26, 27]. In addition, it can protect proteins from proteasomal degradation, most likely through rivalry with the ubiquitination machinery for the same lysine residue [27]. The process of SUMOylation is mechanistically related to ubiquitination, as an enzyme cascade consisting of a SUMO-activating enzyme (E1), a SUMO-conjugating enzyme (E2) and a SUMO-ligase (E3) controls the process of SUMOylation of target proteins. Until today several SUMO-ligases have been identified including PIASx*α*, PIASx*β*, PIAS1, PIAS3, PIASy, RanBP2 (Ran-binding protein 2), Pc2 and HDAC4 (histone deacetylase 4) [reviewed in: 26, 27]. In contrast to ubiquitination, a SUMO-ligase is not obligatory for SUMOylation but some mammalian SUMO-specific E3s stimulate the conjugation reaction, eventually even in a substrate independent manner [28]. The vast majority of SUMO substrates are directly recognized by the SUMO-conjugating enzyme, Ubc9, that binds to the consensus sequence ΨKxE/D (where Ψ is a large hydrophobic amino acid) [29, 30]. However, polymers of both, SUMO-1 and SUMO-2/3, have also been found at lysines that do not comply with the consensus motif [31, 32]. Since the SUMO-conjugating system utilizes only a single enzyme for the recognition and conjugation processes, a further level of regulation must be provided. This is frequently given by an extension of the recognition motif that may contain phosphorylated (PDSM; phosphorylation-dependent SUMOylation motif) or negatively charged (NDSM; negatively charged amino acid-dependent SUMOylation motif) amino acids in addition to the ΨKxE/D motif [33, 34]. Phosphorylation within the PDSM consensus sequence plays an important role in regulating SUMOylation of several substrates including p53, heat shock factors, GATA-1 and the myocyte enhancer factor-2 (MEF2A) [33-35]. Today, four isoforms of SUMO (SUMO-1 to SUMO-4) have been identified in mammals and transcripts of SUMO-1, -2 and -3 can be detected in all human and mouse tissues, whereas SUMO-4 expression seems to be restricted to immune tissues and to the kidney [36, 37]. SUMO-4 was initially identified as an intronless gene, encoding a protein that shares 86% amino acid homology with SUMO-2 [38]. SUMO-4 seems to be unable to form covalent isopeptide bonds with substrate proteins and may control protein functions *via* non-covalent interactions [39]. SUMO-2 and SUMO-3 are conjugated in a stress inducible manner [reviewed in: 26]. They are very similar on the amino acid level but share only 47% identity to SUMO-1. In contrast to ubiquitin, SUMOylation usually conjugates only a single SUMO molecule to a substrate. Nevertheless, recent reports also implicated the formation of SUMO-chains [31, 40]. Like ubiquitination, SUMOylation can be reversed. SUMO-hydrolyses of the SENP family (sentrin- or SUMO-specific protease) remove SUMO molecules from SUMOylated substrates [reviewed in: 27].

The 81 amino acid polypeptide NEDD8 (neural-precursor-cell-expressed developmentally down-regulated 8) is a relatively uncharacterized member of the family of ubiquitin-like proteins [reviewed in: 41]. Nevertheless, genetic approaches have exposed an obvious role for NEDD8 in cell proliferation, viability and development although the number of known substrates is rather small. Like ubiquitination and SUMOylation, the conjugation of NEDD8 to its substrate is initiated by an activating enzyme, followed by the activity of an E2 enzyme and an E3 ligase [reviewed in: 41]. In line with the other modifications, the process of NEDDylation is very dynamic and NEDD8 can be removed e.g. by the evolutionarily conserved COP9 signalosome [reviewed in: 42].

## DNA DAMAGE RECOGNITION AND INITIAL SIGNALLING EVENTS

Once a DSB is generated, the information about this damage needs to be propagated to downstream effector machineries such as repair proteins, cell cycle checkpoints and cell death pathways. Accordingly, recognition of the lesion is the first and initial step in the DNA damage response. In a second step, the information about the lesion can then be transported across the damaged cell. Each specific lesion of the DNA is recognized by one or a set of individual DNA damage recognition factors that are associated with certain signaling components. Double strand breaks are generally recognized by the ataxia-telangiectasia-mutated (ATM) and by the DNA-damage-dependent protein kinase (DNA-PK).

**Fig. (1):**
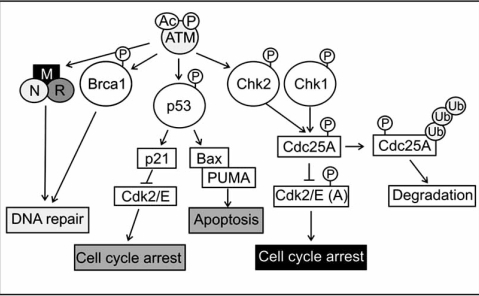
**ATM and its target proteins.** In the active state ATM phosphorylates target proteins that signal to cell cycle checkpoints, DNA repair proteins or to the cell death machinery. Phosphorylation of Brca1 or the activation of the MRN complex results in the initiation of DNA repair. Activation of cell cycle checkpoints that lead to cell cycle arrest are achieved by phosphorylation of the p53 tumor suppressor protein or the Chk2 kinase. In addition, by transcriptional induction of pro-apoptotic proteins like Bax and PUMA, p53 can also induce apoptosis.

**Fig. (2):**
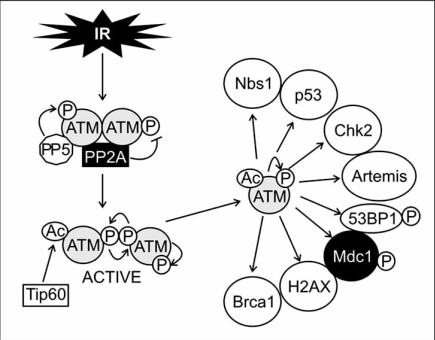
**ATM activation and downstream signaling.** (A) ATM is present in the nucleus as an inactive dimer/multimer that is associated with PP2A, a phosphatase that controls ATM phosphorylation. Upon ionizing radiation (IR), ATM is activated by trans-autophosphorylation and dissociation of the dimer/multimer into monomers as well as by Tip60-mediated acetylation, which enables ATM-mediated phosphorylation of mediator and DNA repair proteins.

ATM is a serine/threonine protein kinase with a multitude of activities. It signals the presence of DSBs to the cell cycle machinery where it mediates proliferation arrest at G1/S, intra-S and G2/M checkpoints (Fig. **1**). The kinase is furthermore required for DNA repair and contributes to the initiation of apoptosis (Fig. **1**) [reviewed in: 43]. Mutations in the ATM gene lead to the genetic disorder ataxia-telangiectasia (AT), which is characterized by cerebellar degeneration, immunodeficiency and an increased risk of cancer. Cells from AT patients are hypersensitive to ionizing radiation and display an increased frequency of chromosome breakage and defects in cell cycle control [reviewed in: 43]. ATM is primarily a nuclear protein where it is present as an inactive dimer or multimer (Fig. **2**). The kinase is, furthermore, associated with protein phosphatase 2A (PP2A), a serine/threonine phosphatase that associates with ATM *via* its scaffolding A-subunit [44]. PP2A dephosphorylates ATM constitutively, which keeps the kinase in an inactive state [44]. In addition to PP2A, ATM was found associated with protein phosphatase 5 (PP5). This interaction of ATM with PP5 increases after exposure of cells to DNA damaging agents and appears to be important for ATM activity in response to DSBs [45]. How ATM becomes activated in the presence of DSBs is not entirely solved. A previous model suggested that upon sensing of a DSB, ATM becomes partially active, resulting in trans-autophosphorylation of ATM at serine 367, serine 1893 and serine 1981, and dissociation of ATM dimers/multimers into highly mobile and enzymatically active monomers that associate with damaged chromatin [46, 47]. More recently, this model of ATM activation has been questioned. Jean Gautier and co-workers showed that the MRN complex and DNA are sufficient to facilitate ATM monomerisation [48]. In addition, phosphorylation of serine 1981 appears to be optional for the dissociation of ATM or its function in general [49]. ATM even remained functional when additional autophosphorylation sites (serine 367 and/or serine 1893) were mutated [50]. Besides phosphorylation, ATM also becomes acetylated in response to DSBs. Tip60 (HIV-1 Tat-interacting protein 60 kDa) acetylates ATM on lysine 3016 and this modification is essential for ATM activation and dissociation into monomers [51]. It is, though, unclear by which mechanism Tip60 becomes activated in the presence of DSBs. Also Aven, an interaction partner of the anti-apoptotic protein Bcl-X_L_, has been reported to contribute to ATM activation [52]. Aven interacts with ATM in cellular extracts and overexpression of Aven promotes ATM phosphorylation on serine 1981 as well as phosphorylation of typical ATM substrates like p53 and Chk2 [52]. However, since Aven is less efficient than DSB in inducing ATM autophosphorylation it appears to act synergistically with DNA damage rather than being a sole activator of the kinase.

Apart from ATM is DNA-PK (DNA-dependent protein kinase) able to recognize DSBs [53]. Like ATM, DNA-PK has a strong affinity to DNA. Its recruitment to sites of DNA damage is conducted by a heterodimer of the Ku70 and Ku80 proteins [54]. The Ku70/80 heterodimer forms a ring-shaped structure that can be threaded onto DNA ends [54]. Due to its high affinity for loose DNA ends and its capacity to bind DNA in a sequence-independent fashion, the Ku70/80 heterodimer is believed to serve as a DSB sensor. Once the Ku70/80 heterodimer is bound to damaged DNA, it serves as a docking site for the catalytic subunit of the DNA-dependent protein kinase (DNA-PKcs), which results in the formation of the DNA-PK holocomplex that displays serine/threonine kinase activity towards p53, H2AX, Artemis, XLF and others [55-58]. Formation of the heterodimeric holocomplex is mediated by the amino-terminal and the central region of both, the Ku70 and Ku80 protein. The Ku80 carboxy-terminus is, furthermore, required for DNA-PKcs autophosphorylation at threonine 2609 [59]. This phosphorylated form of DNA-PK co-localizes with γ-H2AX and 53PB1 at the DSB [59].

**Fig. (3):**
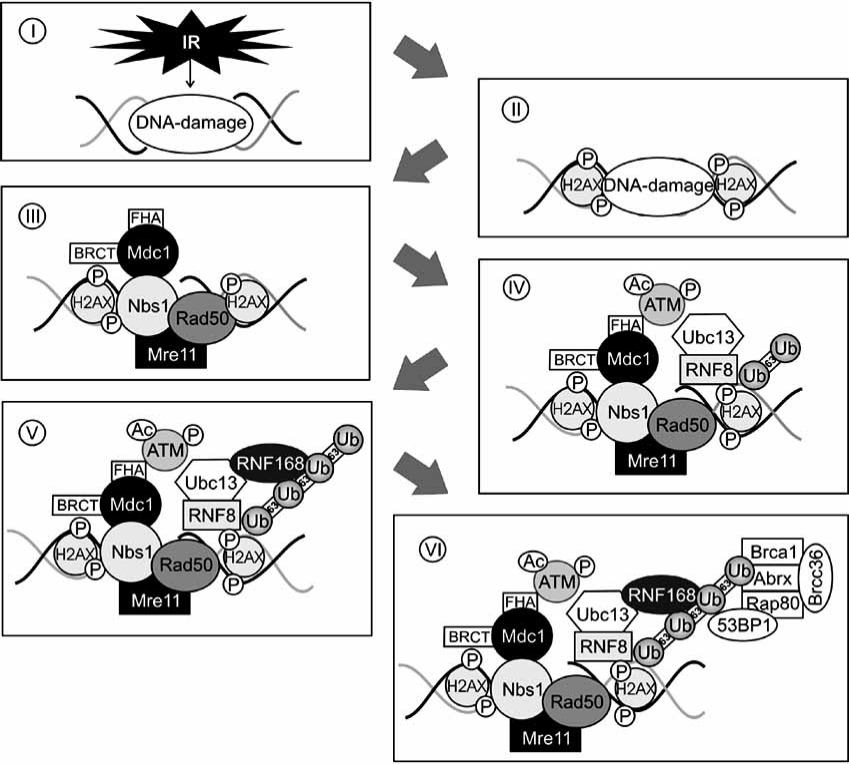
**DNA lesion recognition and recruitment of repair factors.** After introduction of a DNA double strand break (**I**), the DNA damage response is initiated by phosphorylation of H2AX (**II**), which is required for the recruitment of mediator proteins such as MDC1 or the MRN complex. MDC1 attaches to phosphorylated H2AX *via* its BRCT domain and stabilizes the MRN complex (**III**). Additionally it is responsible for binding and accumulation of ATM at the DSB, which creates a docking site for RNF8/Ubc13 that also associates with phosphorylated H2AX and decorates histone proteins with lysine 63-linked ubiquitin molecules (**IV**). RNF168 recognizes the initial ubiquitin chains generated by RNF8 and, in association with Ubc13, extends and propagates lysine 63-linked ubiquitination (**V**) that is required for the accumulation of additional repair factors like Bcra1 or 53BP1 (**VI**).

After recognition of the lesion, an operation platform for repair factors needs to be generated. Since eukaryotic DNA is organized in tightly packed nucleosomes, these structures need to be widened to allow recruitment of repair proteins and to enable their interaction with damaged DNA. For this purpose, different residues of certain histone proteins become post-translationally modified. Especially acetylation, methylation, phosphorylation and ubiquitination of histone proteins have been observed during the DNA damage response [60-65]. The main purpose of acetylation and phosphorylation seems to be to neutralize the positive charge of basic histone proteins. This would reduce the strength of the interaction of basic histone proteins with negatively charged DNA and may decondensate the chromatin. The earliest post-translational modification event at the chromatin as well as for the initiation of the DNA repair process is phosphorylation of the histone H2A variant H2AX at serine 139. The modified H2AX protein, which is called γ-H2AX, appears within a few minutes after exposure of cells to ionizing radiation at the break [64]. Beside H2AX is histone H3 post-translationally modified after a DSB. Within a few minutes after introduction of a DSB, H3 becomes methylated at lysine 79 [66]. Both histone modifications are required for recruitment of mediator proteins such as 53BP1 (p53-binding protein1), MDC1 (mediator of DNA damage checkpoint protein 1) or the MRN (Mre11, Rad50, Nbs1) complex to the DSB [66-69] (Fig. **3**).

The MRN complex, a highly conserved protein complex consisting of Mre11, Rad50 and Nbs1 (Nijmegen breakage syndrome 1), is implicated in DNA repair, cell cycle checkpoint control, DNA replication and telomere maintenance [70-72]. The three proteins, Mre11, Rad50 and Nbs1, form a tight complex which is homogenously distributed throughout the nuclei of mammalian cells but migrates rapidly to DSBs after ionizing radiation, independent of the phase of the cell cycle [73]. The Nbs1 protein appears to be responsible for nuclear localization and the proper assembly of the complex at DSB sites, probably by interacting with phosphorylated H2AX [68]. Mre11 displays endo- and exonuclease activity and is important for the processing of the broken DNA ends [74]. Rad50 also shows DNA binding capacity that possibly plays a role in tethering sister chromatids during HR [75, 76]. The MRN complex is composed of a single Nbs1 molecule that is associated with two dimers of Mre11 and Rad50. The Mre11 and Rad50 proteins themselves form a heterotetramer consisting of a dimer of Mre11 and Rad50. This heterotetramer contains two DNA-binding and processing domains that can bridge free DNA ends [76, 77]. The Mre11 dimer binds and holds the dsDNA ends in close proximity, near the Mre11 active site and it is thought that it aligns and bridges these loose DNA ends during DNA repair [78, 79]. Rad50 contains two long coiled-coil arms that promote intermolecular interactions through a terminal hook domain [76, 77]. Loss of Nbs1 or Mre11 is embryonic lethal [80, 81] and mutations in NBS1 and MRE11 lead to the chromosomal instability disorders Njimegen breakage syndrome (mutation in NBS) and ataxia telangiectasia-like disorder (ATLD; mutation in Mre11). Both of these syndromes are associated with enhanced sensitivity to ionizing radiation and chromosomal instability [reviewed in: 82,  83]. MRN binds to DSBs and leads to further activation of ATM [48, 70]. Accordingly, cells derived from patients with Nijmegen breakage syndrome or ataxia telangiectasia-like disorders exhibit decreased ATM kinase activity despite the presence of wild type ATM [70, 84-86]. The nuclease activity of Mre11 appears to be particularly important for this process [70, 87]. Despite the similar timing of the appearance of Ku70/80 and the MRN complex at DSBs, recruitment of MRN and Ku seems to be independent of each other [88]. Nbs1 and Mre11 are both targets for ATM and at least phosphorylation of Nbs1 is required for checkpoint signaling during S-phase [89]. Therefore the MRN complex acts on the one hand as a downstream mediator of ATM but is also important for the activation of ATM and phosphorylation of downstream substrates.

MDC1, also called NFBD1 (nuclear factor with BRCT domains protein 1), appears to be a master regulator of the microenvironment at damaged chromatin. The docking of MDC1 to DSBs allows retention of multiple checkpoint and adaptor proteins including Nbs1, 53BP1 or Brca1 (breast cancer 1) at the site of the lesion where they provide a molecular platform for efficient amplification of the DNA damage signal [68, 69, 90]. For this, MDC possesses two classes of phospho-binding motifs, a FHA (forkhead-associated) and a BRCT (Brca1 carboxy-terminal repeat) domain that serve as binding partners for phosphorylated proteins. BRCT and FHA modules are conserved throughout different species and are present in many proteins that are involved in the cellular response to DNA damage [reviewed in: 91,  92]. Two repeats of the BRCT domain are located at the carboxy-terminus of MDC1. These BRCT domains associate directly with phosphorylated H2AX and this interaction seems to be essential for the recruitment and docking of MDC1 to the site of the DNA lesion (Fig. **3**). Accordingly, in H2AX^-/-^ MEFs, MDC1 fails to accumulate at DSBs [90]. The FHA domain is positioned at the amino-terminus and binds to ATM which results in the accumulation of ATM at DSBs and enhanced phosphorylation of H2AX and of MDC1 itself [93]. In MDC1-deficient cells, recruitment of ATM to DSBs is impaired [93]. The central domain of MDC1 contains 14 repeats of a sequence that mediate its interaction with DNA-PKcs and the Ku heterodimer [94]. Once recruited to DSBs, MDC1 stabilizes the MRN complex that is bound to damaged DNA and acts as a molecular scaffold for the recruitment of 53BP1, BRCA1 and additional MRN molecules to nuclear foci [68, 90, 95]. Upon binding to MDC1, ATM phosphorylates the mediator protein and this phosphorylation creates a docking site for the recently identified E3-ligase RNF8 (ring finger 8) and for Ubc13 (ubiquitin-conjugating 13; Fig. **3**) [22, 96]. These proteins associate with γ-H2AX and phosphorylated MDC1 *via* their FHA domains and decorate the histone protein H2A and its variant H2AX with lysine 63-linked polyubiquitin chains [22, 96]. Nevertheless, although RNF8 seems to be the first E3 ligase at the DSB, it appears to be insufficient for sustained ubiquitination of the chromatin. It probably rather primes the chromatin around the DSB, which then facilitates the recruitment of another E3 ligase, the RNF168 protein (Fig. **3**) [97]. RNF168 recognizes the initial ubiquitin chains generated by RNF8 *via* its ubiquitin-binding domain and, in concert with Ubc13, propagates and extends the formation of lysine 63-linked ubiquitin chains of histone proteins. Eventually, a threshold of lysine 63-polyubiquitin chains may be required to recruit and hold additional repair factors at the DSB. Particularly recruitment of 53BP1 and Brca1 are assumed to depend on such an “interaction trap” made of polyubiquitinated lysine 63 chains (Fig. **3**) [97]. After polyubiquitination of the chromatin, Brca1, a tumor suppressor protein that also possesses ubiquitin ligase activity, especially when it is complexed with Bard1, is recruited to the lesion together with Rap80, Abraxas, Brca1 and Brcc36, which form the BRCA1-A complex [98]. Brca1 can be found in three different complexes (Brca1 A, B and C), which depend on different adaptor proteins. The adaptor proteins for the complex A, B and C are Abraxas, Bach1/Brip1 (BRCA1-associated C-terminal helicase) and CtIP (CtBP-interacting protein), respectively. Each complex forms in a mutually exclusive manner [98-100]. Whereas complex A plays an important role in DNA damage repair, the Brca1/Bach1 and the Brca1/CtIP complex form at different stages of the cell cycle and are required for a prolonged G2 phase and for G2/M transition checkpoint control, respectively [99, 100]. Ionizing radiation leads to ATM-dependent phosphorylation of Rap80 (receptor-associated protein 80) at serine 140, serine 402 and serine 419 [22, 98]. RAP80, furthermore, possesses two ubiquitin interaction motifs (UIM) which recognize lysine 63-linked ubiquitin chains. These UIMs facilitate the association of the complexes with polyubiquitinated histone proteins [22, 97]. Brcc36 of the complex has ubiquitin hydrolyzing activity and plays an important role in the regulation of the E3 ligase activity of Brca1 in response to ionizing radiation [101, 102]. The Rap80/Brcc36 complex furthermore removes lysine 63-linked polyubiquitin chains from the chromatin and is thus also involved in the termination of RNF8-Ubc13-mediated polyubiquitination once the lesion has been repaired [103]. Although primarily implicated in HR, there is accumulating evidence that Brca1 may also be involved in NHEJ. The N-terminal region of Brca1 specifically associates with Ku80 and rapidly accumulates at DSBs in a Ku-dependent manner. Brca1 is furthermore assumed to control the fidelity of NHEJ [104, 105].

53BP1 functions, together with MDC1 and Brca1, as an adaptor/mediator protein of the DNA damage response. As such, it contributes to the activation of downstream effector molecules that function in DNA repair and DNA damage signaling, although it has no enzymatic activity. 53BP1 was originally identified due to its ability to bind to p53 [106]. Upon ionizing radiation, it accumulates in foci at DSBs and becomes phosphorylated at several sites in an ATM-dependent manner. Among these is phosphorylation of serine 1219 particularly important as phosphorylation of this site assists in recruitment of MDC1 and γ-H2AX [107]. 53BP1 is furthermore required for p53 accumulation, G2/M and intra-S-phase arrest, ATM autophosphorylation at serine 1981 and for the formation of Brca1-containing foci in response to ionizing radiation [108, 109].

## DOUBLE STRAND BREAK REPAIR AND SIGNAL TRANSDUCTION

**Fig. (4):**
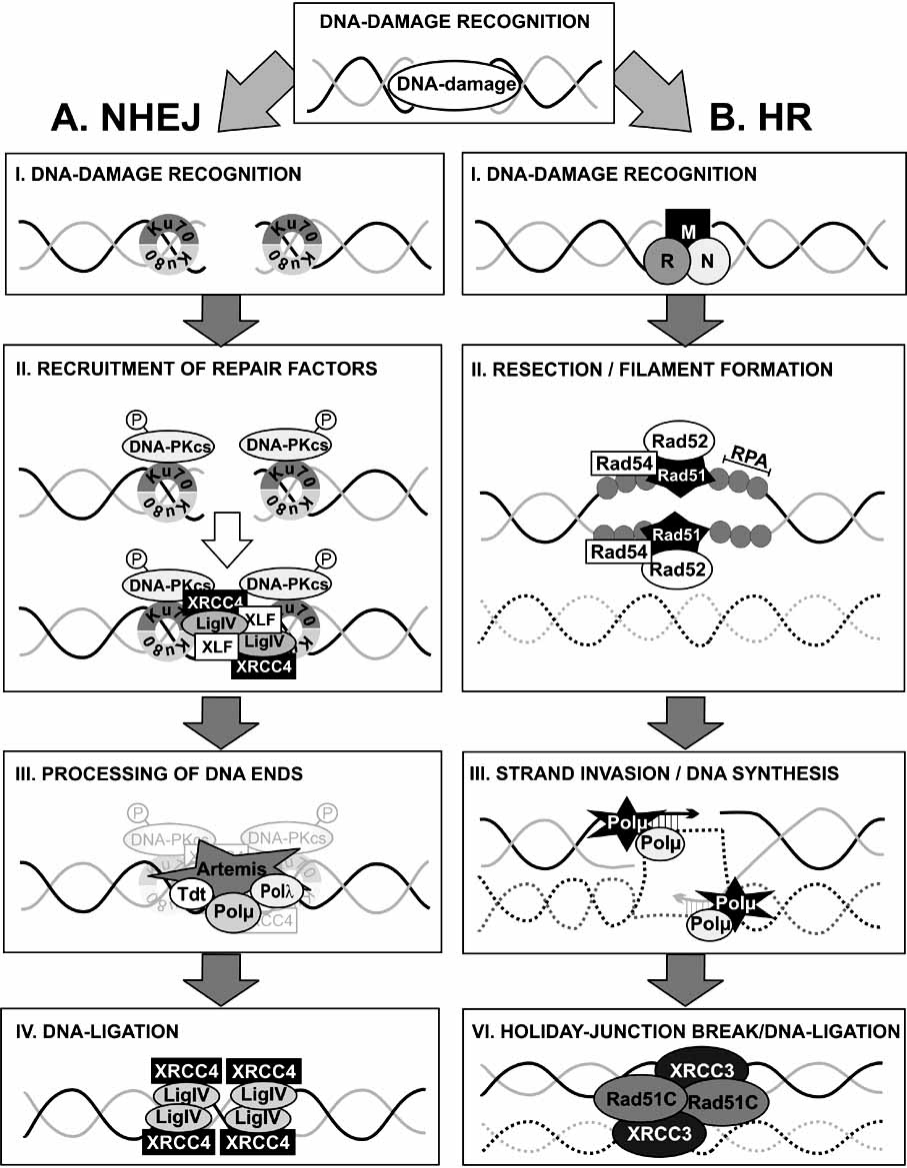
**Mechanisms of DNA-repair by NHEJ and HR.** Double strand breaks are repaired by two major pathways: non-homologous endjoining (A, NHEJ) and homologous recombination (B, HR). **NHEJ**: Loose DNA-ends are recognized by the Ku70/80 heterodimeric complex (**A.I**), which is needed for the recruitment of the additional repair factors DNA-PKcs, XRCC4, LigIV and XLF (**A.II**). Before broken DNA ends can be ligated, the ends are processed by Pol µ and λ, Artemis and Tdt (**A.III**). This step is followed by the ligation of the loose ends by LigIV and XRCC4 (A.IV). HR: HR is initiated by the MRN complex (**B.I**), followed by the recruitment of RPA that associates with single stranded DNA, and by Rad51/52 which are needed for filament formation and strand invasion (**B.II**). New DNA is synthesized while the homologous sister chromatid serves as a template (**B.III**). After branch migration, the holiday junction is resolved (**B.IV**).

After the generation of a protein interaction platform, repair factors are recruited to the DSB that ligate the broken DNA ends. In mammalian cells DSBs are usually repaired by non-homologous endjoining (NHEJ) and homologous recombination (HR) (Fig. **4**). In dependence on the phase of the cell cycle the one or the other mechanism dominates. Nevertheless, while it was previously suggested that during the G1 and early S phase of the cell cycle, NHEJ is the pathway of choice and HR would only be operative in late S and G2, it becomes more and more evident that at least NHEJ is active in all phases of the cell cycle [110, 111]. This is, for example, evidenced by the appearance of Ku70/80, DNA-PKcs and ATP-dependent DNA ligase IV (LigIV) at DSBs not only in G1 but also in S and G2 phase. Nevertheless, although proteins of both repair pathways appear at the same DSB, assembly of the NHEJ machinery clearly precedes that of HR factors, even in the S and G2 phase of the cell cycle [88, 112, 113]. Moreover, the fact that factors of both repair machineries are present at DSBs at the same time suggests that NHEJ and HR are not two competing parallel pathways but rather that NHEJ may serve as an immediate early repair event whereas HR may repair persisting DNA lesions [88].

### Non-Homologous Endjoining (NHEJ)

NHEJ is the repair pathway of choice of non-dividing and of mitotic cells during G1 and early S-phase of the cell cycle. Since most cells of higher organisms are in a quiescent state, NHEJ is considered to be the major DSB repair pathway in mammals. The process of NHEJ is characterized by a “simple” ligation of broken ends without the requirement for larger sequence homologies. However, since the creation of ligatable DNA ends usually comes along with loss of genetic information, it is also an error-prone DNA repair mechanism.

The mechanism of NHEJ covers mainly four consecutive phases (i) recognition of the DNA lesion, (ii) sequential recruitment of repair factors, (iii) processing of DNA ends to yield ligatable termini and (iv) sealing of the break. To date seven proteins have been described to be required for NHEJ: the Ku70/Ku80 heterodimeric complex, DNA-PKcs, LigIV, XRCC4 (X-ray repair complementing group 4 protein), XLF (XRCC4-like factor) and Artemis (Fig. **4**) [114-121]. If a cell is defective in one of these enzymes, neither DNA repair nor V(D)J recombination, a cut-and-paste mechanism that uses NHEJ to seal DSBs that arise during the generating of the diversity of antigen receptors in immune cells [reviewed in: 122], can be carried out. This results in enhanced sensitivity towards ionizing radiation and in immunodeficiency [114, 116, 120, 121, 123].

The Ku70/80 heterodimer serves as a recognition and first docking molecule at DSBs and it most likely also protects DNA ends from nucleolytic degradation (Fig. **4**) [114, 124, 125]. *In vivo* studies with Ku-deficient yeast strains demonstrated that Ku is important for efficient NHEJ and for accurate rejoining of complementary and non-complementary DNA ends [114]. The recruitment of Ku70/80 is followed by the arrival of DNA-PKcs at the DSB and its autophosphorylation (Fig. **4**). Mutation of the autophosphorylation site of DNA-PKcs impairs rejoining of broken DNA [59]. After the arrival of DNA-PK, the DNA at the break is processed. DSBs frequently lead to damaged bases or incompatible single strand overhangs at the break, which are unsuitable for direct ligation. Several enzymes with single-strand filling capacity or nucleolytic trimming potency have been suggested to play a role in the processing of the DNA, like polymerase λ, polymerase μ, Tdt (terminal deoxynucleotidyltransferase) and Artemis (Fig. **4**). These enzymes add or remove nucleotides or extend the 3´-single stranded DNA tail in a template-independent manner. The DNA polymerases λ and μ are responsible for refilling the gaps that result from NHEJ intermediates while Tdt adds nucleotides to loose ends during V(D)J recombination [126-128]. Artemis, on the other hand, displays an intrinsic 5´-3´exonuclease activity *in vitro* and assists in the repair of a subset of DSBs [129]. Upon forming a complex with DNA-PKcs, Artemis becomes phosphorylated, which facilitates cleavage of 5´and 3´overhangs, nicks and hairpin structures at the loose end of the DNA [129]. Artemis is furthermore important for the opening of hairpin structures during V(D)J recombination [129].

LigIV is a core component of the NHEJ repair pathway and catalyzes the ligation of the DNA ends (Fig. **4**). Structural studies have revealed that LigIV exists in a tight complex with XRCC4 that is accomplished by the central region of XRCC4 and that strongly enhances LigIV stability [117, 130, 131]. XRCC4 is a nuclear protein that is constitutively phosphorylated and its phosphorylation is further enhanced in response to ionizing radiation in a DNA-PK-dependent manner [132]. In addition to phosphorylation, a monoubiquitinated form of XRCC4 has been identified. The abundance of this modified form is enhanced when cells have been treated with etoposide, a topoisomerase inhibitor that generates DSBs [133]. The precise role of this ubiquitination of XRCC4 has, though, not been determined. In addition to phosphorylation and ubiquitination, XRCC4 is modified with SUMO at lysine 210, a modification that appears to be required for nuclear localization of the protein [134].

More recently XLF (XRCC4-like factor), also called Cernunnos, was identified as a new interaction partner of XRCC4 and an additional member of the NHEJ machinery [118, 119]. XLF forms homodimers with a XRCC4-like structure and modulates XRCC4/LigIV activity through direct interaction with XRCC4 [135, 136]. XLF is quickly recruited to DSBs in a Ku-dependent and XRCC4-independent manner [137]. Although XLF is phosphorylated by DNA-PK at serine 245 and by ATM at serine 251, no particular function could be attributed to these modifications [58].

Once repair is completed, the repair machinery needs to be disassembled, but how this is achieved has not yet been understood in detail. Only for the Ku70/80 proteins some insights into this process have been provided. Recently it was demonstrated that Ku80 bound to DSB is heavily modified with polyubiquitin chains. This modification appears to mediate efficient removal of Ku80 from repaired DNA [138]. Most interestingly, binding of Ku to DNA appears to initiate its polyubiquitination, which then allows its removal from the DNA once the lesion is fixed [138].

While the classical NHEJ pathway uses the XRCC4/LigIV complex to ligate broken DNA ends, the immune system can make use of an alternative NHEJ pathway that is XRCC4-independent [139]. This alternative NHEJ mechanism deletes sequences at the loose ends until short stretches of homology appear at the repair junction. These areas of microhomology assist in ligation in a process called microhomology-mediated endjoining (MMEJ). It is most likely that the enzymatic and scaffolding function of Mre11 participates in this process [140]. Whether this alternative route of NHEJ also contributes to the sealing of DSBs after ionizing radiation, e.g. when XRCC4 is absent, remains to be determined.

### Homologous Recombination (HR)

HR is particularly activated during late S- and G2-phases of the cell cycle as this repair pathway requires sequence homologies of about 100 base pairs and more in close proximity to the damaged site. These conditions are normally only provided between DNA replication and cell division. Since the undamaged sister chromatid is used as a template, this type of repair is generally accurate. As HR requires the alignment of broken DNA ends with a homologous region of the genome, homology search and DNA strand invasion is a central component of HR. After introduction of a DSB, HR is generally initiated by nucleolytic resection of the broken DNA which results in long single stranded tails with 3´-hydroxyl overhangs. It is most likely that the Rad50/Mre11 complex contributes to this activity [78]. The resulting single stranded DNA (ssDNA) is bound by the replication protein A (RPA) (Fig. **4**) [141]. RPA is a heterotrimeric, eukaryotic protein that binds to ssDNA in a sequence independent manner thus forming a nucleoprotein filament [reviewed in: 142]. Apart from protecting the ssDNA from nucleolytic attacks, RPA eliminates secondary structures, which allows more Rad51 molecules to access and bind ssDNA, thus stimulating the formation of a presynaptic complex of DNA, RPA and Rad51. RPA can have positive and negative effects on Rad51-mediated strand exchange. Pre-incubation of ssDNA with RPA protein prevents binding of Rad51 to the DNA but this inhibitory effect can be diminished by Rad52 or Rad55/57. The Rad55/57 complex interacts transiently with Rad51 which results in the abolishment of the RPA inhibitory effect and support of the formation of the Rad51 filament, similar to Rad52 [143]. In contrast, when RPA is present after the Rad51/DNA complex has formed, it is able to stimulate strand exchange *via* Rad51, possibly by removing secondary structures of the ssDNA, and to promote appropriate Rad51 filament formation [141, 144].

The main activity of Rad51 is to promote ATP-dependent strand exchange, catalyzed by an intrinsic ssDNA-dependent ATPase activity [141, 145]. Accordingly, yeast cells lacking Rad51 are hypersensitive to ionizing radiation and alkylating agents [141, 146]. In humans, five Rad51 paralogs exist named Rad51B, Rad51C, Rad51D, XRCC2 and XRCC3 [reviewed in: 147]. The mammalian counterpart of Rad52 is Brca2, a protein frequently mutated in breast and ovarian cancer. Similar to the yeast counterpart, Brca2 controls the activity of the recombinase, Rad51, and stimulates the loading of Rad51 onto ssDNA [148-150]. Once Rad51 has been recruited to the nucleoproteinfilament, it replaces RPA. The 3´-overhangs of the DNA associate with Rad52 and subsequently with polymerized Rad51 and form the so-called recombinase filament where Rad51 and Rad52 are wrapped around the ssDNA overhang. This interaction is stabilized by Rad54 that associates with the recombinase filament and enhances their catalytic activity [151]. Once this complex is complete, the tail of Rad52 starts to search for a homologous sequence in the sister chromatid and facilitates base pairing with the single-stranded overhang to form a D-loop intermediate. This homology search is furthermore assisted by Rad51 which, together with the ssDNA, invades into the intact homologous chromosome to form a heteroduplex. Rad54 appears to help in this process, although its precise function remains to be determined [152]. After formation of the heteroduplex, DNA polymerase µ and λ extend the 3´end of the invading strand in a process called branch migration (Fig. **4**) [153]. Due to strand invasion, a joint molecule with crossed-over DNA strands is generated. To finish the repair process, these holiday junctions need to be resolved in order to reconstruct the two individual DNA double helices. The resolution of the holiday junction is performed by the resolvases Rad51C and XRCC3 (Fig. **4**) [153-156].

## CELL CYCLE ARREST AND APOPTOSIS

In mammalian cells, evolutionary conserved pathways exist that signal information about the state of the genome to a series of checkpoints, which can delay the progression into the next phase of the cell cycle. All these checkpoints are tightly regulated. In response to DNA damage, errors in DNA replication or chromosome segregation, cells can activate these checkpoints to allow time to fix the lesion before the cell reinitiates replicative DNA synthesis or begins mitosis. The most important checkpoints within the cellular response to DNA damage are the G1/S and the G2/M checkpoints. In addition, an intra-S-phase checkpoint exists [reviewed in: 157].

After sensing of the DSB, the information about the lesion is rapidly transduced to the checkpoints of the cell cycle. ATM and Chk2 (cell cycle checkpoint kinase 2) kinases play an important role in this process [158-160]. Chk2 is a stable protein which is expressed throughout the cell cycle. In the absence of DNA damage Chk2 is largely inactive. When DNA damage occurs, the kinase becomes rapidly phosphorylated at threonine 68 by ATM [158]. This phosphorylation stimulates Chk2 homodimerisation and activates the kinase, which results in autophosphorylation at threonine 383, threonine 387 and serine 516, and phosphorylation of Chk2 target proteins [159-161]. One of the targets of Chk2 is Cdc25A, a phosphatase that dephosphorylates and activates cyclin-dependent kinases (Cdks) (Fig. **1**) [162]. These kinases drive the cell trough the cell cycle. Phosphorylation of Cdc25 by Chk2 in response to DSBs leads to rapid proteosomal degradation of the phosphatase and thus to inactivation of cdk2/cyclin E/A complexes as well as of cdk2/cyclin B/A complexes, which result in cell cycle arrest at the G1/S and G2M checkpoint, respectively [162, 163].

An additional important component of checkpoint activation in response to DNA damage is the p53 tumor suppressor protein (Fig. **1**). Most characteristic for p53 are its proliferation-inhibiting and pro-apoptotic activities. Many, but not all of these activities are caused by its function as a transcription factor. Under normal growth conditions, the amount of the p53 protein is maintained low and the protein is transcriptionally inactive. Both parameters are mainly controlled by the E3 ubiquitin ligase Mdm2, although other proteins may also contribute to this process [reviewed in: Boehme and Blattner,  in press]. Mdm2 binds to the N-terminal transactivation domain of p53 and mediates its ubiquitination and proteosomal degradation [164]. Upon DNA damage, p53 is protected from degradation and post-translationally modified at several sites in its amino- and carboxy-terminal part, which enhances its sequence-specific binding to promoters of target genes and activation of gene transcription [12, 165-168]. Among the target genes of p53 are *bax*, *puma* and *p21*, while transcription of other genes, such as *bcl-2* are repressed [169-171, reviewed in: 172]. p21, also known as p21^WAF1/Cip1^ is a member of the Cip/Kip family of cyclin-dependent kinase inhibitors [reviewed in: 173]. Cyclin dependent kinases 2, 4 and 6 (Cdk2, Cdk4, Cdk6) and the associated cyclins, control progression through the cell cycle by phosphorylation of their target proteins [174]. An important substrate of theses cyclin/Cdk complexes is the retinoblastoma protein (Rb), a transcriptional repressor of the E2F family of transcription factors. E2F proteins control entry into and progression through the S-phase of the cell cycle. In its hyperphosphorylated state, Rb binds to E2F family members and prevents them from activating their target genes, which, at the same time, precludes entry into the S-phase of the cell cycle [175, 176]. In unstressed cells, Rb becomes phosphorylated at the end of S-phase by cyclin D1/Cdk4,6, which leads to the dissociation of E2F and Rb and transcription of E2F target genes [177]. Upon exposure to genotoxic agents, the Cdk-inhibitor p21 is induced and abolishes Cdk-mediated phosphorylation of Rb, which results in hypophosphorylation of Rb, sequestration of the E2F protein and cell cycle arrest at the G1/S boundary of the cell cycle [171].

**Fig. (5):**
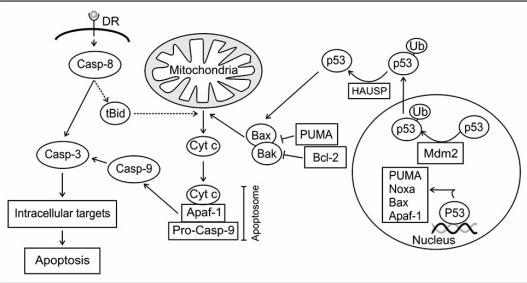
**Apoptosis signaling pathways**. Apoptosis can be induced upon activation of death receptors (DR) at the membrane by binding of cognate ligands or after release of pro-apoptotic factors from mitochondria. Upon DR activation, the initiator-caspase-8 becomes activated, which activates a caspase-cascade, and cleaves the pro-apoptotic proteins Bid. The cleavage of Bid results in the formation of active tBid, which, in cooperation with Bax and Bak, forms pores in the outer mitochondrial membrane resulting in the release of cytochrome c, formation of the apoptosome and activation of caspase 9. Caspases 9 activates caspase 3 leading to the degradation of intracellular targets. The proapoptotic proteins Bax and Bak can be inhibited by Bcl-2 and Puma. Besides its nuclear function p53 can become monoubiquitinated in a Mdm2-dependent manner, which leads to the translocation of p53 to mitochondria where it participates in mitochondrial membrane permeabilization and cytochrome c release.

If the damage of the DNA is too severe, programmed cell death (apoptosis) can be initiated to eliminate potential hazardous cells, which bear the risk of uncontrollable behavior or transmission of mutations to daughter cells. Apoptosis is a form of cell death that is characterized by specific morphological changes of the cell like overall shrinkage, chromatin condensation and fragmentation of genomic DNA [178]. Apoptosis can be initiated by different extracellular and intracellular factors like UV-light or ionizing radiation, chemotherapeutic drugs, by activation of death receptors or by reactive oxygen species. The apoptotic program can be started by two main pathways, an extrinsic one that is initiated by binding of ligands to membrane-bound death receptors and activation of caspase 8, and by an intrinsic one, that is initiated by mitochondrial membrane permeabilisation, release of cytochrome c from the intermembraneous space and activation of procaspase 9. Both pathways lead to activation of caspase 3, which initiates controlled degradation of the cell (Fig. **5**) [reviewed in: 179].

Mitochondrial membrane permeabilisation is controlled by a number of pro- and anti-apoptotic proteins, most of which belong to the Bcl-2 superfamily. Members of this family fall into three groups and all either stimulate or inhibit programmed cell death. Bcl-2, Bcl-X_L_ and Mcl1 belong to the group of anti-apoptotic proteins while Bax and Bak are pro-apoptotic proteins. The third group is composed of the BH3-only proteins, which can be subdivided into “activators” and “de-repressors”. The activators (e.g. tBid) can directly interact with the pro-apoptotic effector proteins Bax and Bak and induce their oligomerization which leads to the formation of pores in the mitochondrial membrane. The de-repressors (e.g. PUMA) act in a more indirect way on the activation of Bax and Bak as they bind to anti-apoptotic proteins that guard Bax and Bak and force their release from the inactivating complex [reviewed in: 180].

p53 initiates apoptosis in response to DNA damage primarily through transcriptional activation of genes encoding apoptotic factors such as Bax, PUMA or Noxa, which neutralize anti-apoptotic proteins from the IAP family [169, 170, 181]. However, a significant portion of the tumor suppressor has also been found in the cytoplasm, where it is also prevented from degradation and contributes to the initiation of cell death. In response to DNA damage, cytoplasmic p53 induces the oligomerization of Bax, Bak, VDAC and cyclophilin D, which triggers the permeabilisation of the mitochondrial membrane, resulting in the release of the pro-apoptotic molecules cytochrome c, smac and AIF [182, 183]. The mechanism how p53 translocates to the mitochondria is not fully understood. Neither phosphorylation nor acetylation appears to contribute to this process. However, it has been suggested that this process may be triggered by Mdm2-mediated monoubiquitination of p53 [184]. Once p53 has arrived at the mitochondrial membrane it is rapidly deubiquitinated by the local mitochondrial HAUSP protein *via* a stress-induced mitochondrial p53-HAUSP complex, creating the apoptotically active, non-ubiquitinated p53 protein [184].

## CONCLUSIONS

The whole process of DNA repair, starting with the sensing and recognition of a DNA strand break and finally resulting in the ligation of the loose ends, is accomplished by a very complex network of interacting proteins that is only incompletely understood. Efforts of more recent years show that a bunch of different players are involved in the DNA damage response. Moreover, there is more and more evidence that many of the sensing, signaling and repair factors interact with each other. Other repair factors even contribute to several different signaling and repair pathways. Examples of such multitalented factors are the MRN complex or ATM, but also Brca1, H2AX, PARP-1, RAD18 or DNA-PKcs. But even though this complex network began to become clearer in more recent years, a lot of open questions are left, including the exact sensing mechanism of DNA damage and the identity of all the proteins involved in this process, the communication of the factors with each other and the basics of the choice between different repair pathways.

Defects in the key players of the DNA damage response can result in a very severe outcome like genomic instability and predisposition for cancers. However, while DNA repair is of utmost importance for healthy individuals, it can be counterproductive during cancer therapy, which frequently uses the implementation of DNA damage to kill tumors by apoptosis. Therefore, attempts are currently under way to inhibit DNA repair during cancer therapy by specifically targeting key proteins of the repair machinery. Along this line, several inhibitors targeting DNA-PK or ATM have been synthesized, which are currently tested for their application in humans. A detailed understanding of the DNA repair networks would help to identify further targets and greatly support the development of new drugs for the treatment of cancer.
